# Sign2Pose: A Pose-Based Approach for Gloss Prediction Using a Transformer Model

**DOI:** 10.3390/s23052853

**Published:** 2023-03-06

**Authors:** Jennifer Eunice, Andrew J, Yuichi Sei, D. Jude Hemanth

**Affiliations:** 1 Department of Electronics and Communication Engineering, Karunya Institute of Technology and Sciences, Coimbatore 641114, India; 2 Computer Science and Engineering, Manipal Institute of Technology, Manipal Academy of Higher Education, Manipal 576104, India; 3 Department of Informatics, The University of Electro-Communications, Tokyo 182-8585, Japan

**Keywords:** sign language recognition, gloss prediction, transformer, pose-based approach, pose estimation, deep learning

## Abstract

Word-level sign language recognition (WSLR) is the backbone for continuous sign language recognition (CSLR) that infers glosses from sign videos. Finding the relevant gloss from the sign sequence and detecting explicit boundaries of the glosses from sign videos is a persistent challenge. In this paper, we propose a systematic approach for gloss prediction in WLSR using the Sign2Pose Gloss prediction transformer model. The primary goal of this work is to enhance WLSR’s gloss prediction accuracy with reduced time and computational overhead. The proposed approach uses hand-crafted features rather than automated feature extraction, which is computationally expensive and less accurate. A modified key frame extraction technique is proposed that uses histogram difference and Euclidean distance metrics to select and drop redundant frames. To enhance the model’s generalization ability, pose vector augmentation using perspective transformation along with joint angle rotation is performed. Further, for normalization, we employed YOLOv3 (You Only Look Once) to detect the signing space and track the hand gestures of the signers in the frames. The proposed model experiments on WLASL datasets achieved the top 1% recognition accuracy of 80.9% in WLASL100 and 64.21% in WLASL300. The performance of the proposed model surpasses state-of-the-art approaches. The integration of key frame extraction, augmentation, and pose estimation improved the performance of the proposed gloss prediction model by increasing the model’s precision in locating minor variations in their body posture. We observed that introducing YOLOv3 improved gloss prediction accuracy and helped prevent model overfitting. Overall, the proposed model showed 17% improved performance in the WLASL 100 dataset.

## 1. Introduction

Sign language, which has its own underlying structure, grammar, syntax, and complexities, is the main mode of communication among the *Deaf Community.* To comprehend sign language, one must consider a plethora of factors involving hand movements, head, hand posture, shoulder posture, location of the lips, and facial expressions. However, in an environment where spoken language is much more prevalent, the deaf community faces challenges of communication barriers and separation from society. To alleviate communication difficulties, understanding sign language as a spoken language is becoming incredibly valuable.

The early stages of sign language research focused primarily on sign language recognition (SLR). SLR focuses on action recognition from the performed sign language sequence without paying attention to its grammatical and linguistic structures. In other words, SLR interprets the performed signs of alphabets [1], numbers [2], or symbols [3] from either static images or continuous sequences of images [4] and is categorized into Isolated SLR [5] and Dynamic SLR [6]. Continuous SLR recognizes sign postures from a continuous sequence of sign language videos which can either be an isolated word video or a continuous spoken sentence sequence, whereas isolated SLR recognizes sign postures from a single static image. Prior systems relied on hidden Markov model-based sequence recognition [7] and per-image feature extraction [8]. The effectiveness of automatic voice recognition served as inspiration for this pipeline. The design of the features that needed to be retrieved posed the biggest challenge in SLR. It was challenging to create a reliable algorithm that could extract the key linguistic elements, such as hand form [9], body movements [10], and face expression [11], even though they had already been recognized. Later, with the advancement of deep learning, manually constructed feature extraction was replaced by automatically extracted features using CNN models [2,12,13]. The overfitting, class imbalance, and exploding gradient problems caused them to perform poorly despite carrying out automatic feature extraction. Likewise, they significantly lagged in encoding the object’s orientation and position. Soon, many hybrid models combined with CNN and HMM [14], CNN with DCGAN [15], CNN with LSTM [16,17], CNN with SVM [18], and CNN with hybrid segmentation [19] emerged. The outbreak of 3D CNN [17,20,21] created outstanding growth in spatio-temporal feature extraction.

Although deep learning has produced state-of-the-art results in the various challenges of SLR [16,22], to enhance the training process of the end-to-end sequence translation process, deep learning models require annotated datasets to tune CSLR models. For this to happen, the model should be trained with isolated words to increase the performance of the CSLR models. To resolve this issue, Chen et al. [23] proposed a transfer learning-based approach. This approach addressed data scarcity by gradually pretraining visual and linguistic modules from general domains into the target domains to some extent. This strategy also required annotated data to improve the model’s performance. The development of better-trained sign language translation models is hampered by a lack of data. Owing to this issue, the performance of current CSLR models needs to be improved. Though various methods and architectures have been proposed to address exact interpretations of sign language through SLR and CSLR, there still lacks meaningful translation of the performed sign language. Ever since the advent of deep learning and its application in computer vision, the pairing of vision and language has received a lot of attention.

Sign language translation (SLT) [24] is the transcription of a sign language video to spoken sentence phrases, paying attention to all the rich underlying grammatical structures that allow the user to understand the underlying language model, spatial representations, and the mapping pattern between the sign and spoken language. SLT is far more complex than SLR because it considers additional visual cues such as body posture, facial expressions, and signing position. While performing sign transcription, which is literally a written version of sign performance, glosses are the intermediary representation. Glosses are words associated with a specific sign, also known as a label [25]. The structure of glosses differs from that of spoken languages. They serve as the foundation of complete sign sequence translation. For example, if a signer performs a sign sequence for the phrase “The weather is too cold today”, the sign translation model suggests the relevant glosses, such as “weather”, “cold”, and “today”. In this paper, we focus on enhancing gloss prediction accuracy in isolated SLR by reducing computational and timing complexities. The end goal of SLT is the transcription of sign language into spoken sentences. End-to-end translation and two-stage translation are the two types of SLT. End-to-end translation directly translates the sign video from the sign sequence [26], whereas two-stage SLT generates intermediate glosses from the sign video; from the glosses, the spoken sentence translation is generated while accounting for the underlying rich grammar [27].

Although end-to-end translation requires less work in terms of components and relies on naturally occurring data rather than domain knowledge and specialist linguistics, these models require a large amount of training data to achieve the aforementioned benefits. In contrast, in two-stage translation, the intermediate gloss representation settles out-of-vocabulary issues that frequently occur in end-to-end translation [28]. Therefore, understanding the importance of gloss in SLT, in this work, we concentrate on word-level gloss prediction to enable appropriate gloss prediction in the case of translating continuous sign sequences.

**Figure 1:**
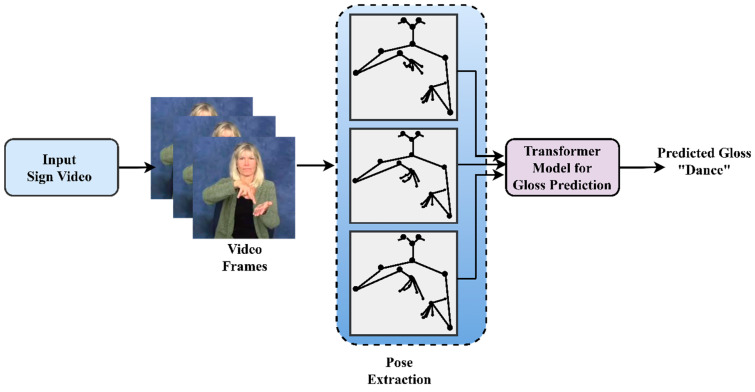
Overview of gloss prediction from sign poses—WLASL using a standard transformer.

In this research, we propose a Sign-Pose2Gloss Prediction Transformer that eliminates the need for expensive and time-consuming labelled data to train the model. Thus, rooting out a mathematical pattern between elements is unnecessary since our model has self-supervised learning abilities. In our approach, considering the real-time challenges faced by the SLT, we suggest a novel pose-based model trained and evaluated on the large-scale word-level American sign language (WLASL) dataset for gloss prediction. In our manuscript, we use the sample gloss figures from asl-lex.org. We acknowledge them with the following citations [29,30]. The model’s input will be dynamic videos made up of sign words, as shown in Figure 1, which explains how the model operates. To distinguish essential frames from redundant frames, we propose the modified histogram difference approach in conjunction with the Euclidean distance algorithm. In comparison to all other pose-based models and frameworks in use, this process made our model more accurate at predicting even similar gloss words. Additionally, we employ hand-crafted augmentation techniques, including in-plane rotation, joint angle rotation, and perspective modification to extracted pose vectors to enable our model to be considerably more adaptable to real-world applications. Furthermore, by learning the location of the target pose vectors using a bounding box, we further prevent our model from overfitting and generalization by utilizing YOLOv3 to normalize the pose vectors.

On the basis of the human pose-based modelling, we emphasize isolated SLR for gloss prediction knowing that recognizing word-level sign itself is exceptionally hard and serves as a basic core element for recognizing continuous sentences in CSLR. We consider that a person’s skeletal motion greatly contributes to the messages they are expressing. Inspired by the transformer architecture proposed by Ashish et al. [31] with slight modifications to the standard transformer model, we evaluate the potential of the proposed transformer model. Transformer models perform remarkably well in tasks requiring sequence processing and are relatively inexpensive computationally.

Our key contributions in pose-based word-level sign language recognition (WSLR) include:

1. We introduce a novel approach for our pose-based WLSR using a keyframe extraction technique to discard the irrelevant frames from the critical frames. To perform this keyframe extraction, we use a modified histogram difference algorithm and Euclidean distance algorithm through which our model achieves 5% improvement compared to other existing pose-based state-of-the-art results on all the subsets of the WLASL dataset (WLASL 100, WLASL 300, WLASL1000, WLASL 2000).
2. We employ augmentation techniques that let our model fit and be adapted for any additional real-time dataset in generalizing so that it can handle the real-time scenario. For this, we adopt in-plane rotation with perspective transformation and joint rotation, which has the added benefit of enabling our model to recognize poses executed at various angles, with various hand sizes, and even at various locations.
3. We introduce a novel pose normalization approach in WLSR using YOLO v3, through which our approach has seen significant improvement of up to 20% for the exact detection of the pose vectors in the signing space.
4. To predict the glosses from the normalized pose sequence, we propose a novel method through a Sign2Pose Gloss prediction transformer, which attains the highest top 1% recognition accuracy of 80.9 in WLASL 100, 64.21 in WLASL 300, 49.46 WLASL 1000, and 38.65 WLASL 2000, surpassing all state-of-the-art outcomes from the existing pose-based models.

The body of the article is structured as follows: Section 2 outlines the prior work in the field of sign language translation and offers insight into the issues that still need to be resolved to considerably increase identification accuracy using pose-based approaches. Section 2.1 discusses the significance and impact of gloss in continuous sign language translation. The two SLT criteria are discussed in depth in Section 2.2 to clarify how two-stage SLT translation overcomes the challenges of end-to-end SLT. Section 2.3 summarizes the importance of video summarization techniques in SLT. Section 2.4 explains how key point generation and pose estimation aid in recognizing finer details in sign sequences for exact gloss prediction. The procedures and approaches for carrying out gloss prediction with our suggested model are described in Section 3. Section 4 provides a quick overview of the proposed Sign2PoseGloss prediction transformer’s detailed architecture. Section 4 discusses the design and validation of the experiment. The performance assessment of our model with an architecture based on appearance and pose is carried out in Section 5, and the results are discussed. Finally, we summarize the research with future scope.

## 2. Related Works

Sign language translation requires visual content and gloss annotation. As discussed in Section 1, the end goal of SLT is to provide natural language spoken sentences. Sign language translation is performed after gloss recognition and further continuous sign language translation. Therefore, in this section, we present the previous literature concepts of deep learning in CSLR models to understand how the role of gloss stands as the backbone for CSLT. To facilitate bidirectional communication between the deaf community and society, building a robust model which would be capable of translating sign language into spoken sentences and vice versa is necessary. Further, we describe the techniques used to address the complexities of video processing and the issues with appearance-based methods in gloss prediction. Furthermore, this study summarizes existing techniques for keypoint extraction and pose estimation models, as well as the requirement for a systematic approach to gloss prediction with reduced processing time complexity. The related works mainly focus on the deep learning-based SLT model to analyze the state-of-the-art results. The following sections summarize the concepts and methods related to SLT.

### 2.1. Significance of Glosses in Vision-Based CSLT

Recognizing the exact gloss representation for the performed sign sequence plays a significant role in CSLT. The biggest challenge of a CSLT system is the insufficient annotated dataset, identifying the explicit boundaries of signed words from the extracted frames of sign video and the transcription of target sentences from the extracted gloss sequences. In the initial phase of work, Hidden Markov models [32,33,34] were widely used for capturing the temporal feature information. Cui et al. [35] proposed a DNN (Deep Neural Network) for temporal feature extraction and RNN for sequence learning. In his framework, he suggested an iterative training process that includes gloss annotations from video segments and an alignment proposal module that generates the aligned sentence sequences from the extracted glosses. It is evident from this approach that the iterative process of sequence learning eliminates the need for massive amounts of information to train an HMM model. Although these modalities are superior in learning temporal dependencies, the integrated approach of multiple modalities necessitates more investigation because the performed sign gestures have concurrently related streams of information. Further, Sharma et al. [36] proposed a deep transfer learning approach employed for sign sentence recognition. In their deep learning-based network model, they used a convolutional neural network along with bi-directional LSTM and connectionist temporal classification (CTC). The added advantage of using this model is it can be trained to recognize the sequence of sentences without any requirement of any prior knowledge in an end-to-end fashion. However, connectionist temporal classification faces severe overfitting during computation. To resolve this issue, Niu et al. [37] used stochastic fine-grain labelling while training the model. For extracting gloss information from sign video frames, the model should know contextual information to extract the actual context of the sign with gloss. To ensure this, Anirudh et al. [38] proposed a pose-based SLR for gloss identification with contextual information using a graph convolutional network (GCN) and BERT transformer. Though this model concentrates on both spatial and temporal fusion extraction, combining a pose-based approach with image-based features will further enhance model performance. On the other hand, Cui et al. [39] proposed a model for real-time CSLR where they used RNN to address mapping issues with relevant glosses by designing a weakly supervised detection network using a connectionist temporal and alignment proposal for continuous spoken sentence translation. Further, this method requires improvement to handle multi-modal information.

To make this easy, transfer learning is employed by initially training the deep learning network using an isolated word dataset so that the problem is addressed. Rastgoo et al. [16] adapted this transfer learning technique using a post-processing algorithm to address the limited labelled dataset issue.

### 2.2. End-to-End and Two-Stage Translation in SLT

With the recent advancement in neural machine translation, recent works have concentrated on designing a gloss-free model to generate textual content directly from visual domains using cross-modal mappings without any intermediate glosses. Zhao et al. [40] proposed a novel framework for sign video to spoken sentence generation using three key modules. In their model, they replaced the gloss generation module with a word existence module that checks the word existence in the input sign video. For this, they applied a CNN encoder–decoder for video feature extraction and a logistic regression classifier for word existence verification. However, in the existing proposed model, there still exist challenges in visual-to-text direct mappings. Additionally, training an SLT model is challenging for longer sentences/video sequences, and decoding a sentence from the input sign video after extracting finite dimensional features is tedious. Further, a key point normalization method to normalize the skeleton points of the signer was proposed by ref. [41] to translate sign videos into spoken sentences directly without any intermediate gloss. They applied the stochastic frame selection method for sampling and frame augmentation and transcribed sign language videos into spoken sentences using attention models. However, direct sign-to-text translation outcomes were no better. Since end-to-end translation requires a huge amount of information to train and tune the model, two-stage SLT is the better option for CSLT; however, it is time-consuming to process the input sequence.

When compared with gloss, mid-level representation drastically improves SLT performance [24]. Additionally, sign-to-gloss translation averts long-term dependencies [42], and the number of sign glosses from a particular sign video are minimal when compared with the number of frames in the video [14]. Therefore, combining gloss representation with recognition and translation of sign language, a unified architecture is proposed by Camgoz et al. [43] that jointly learns continuous sign language recognition and translation achieved by CTC, thereby improvising sequence-to-sequence learning and performance independent of ground truth timing information. The detailed summary of the existing deep learning models for two-stage SLT is discussed in Table 1.

In the same way, sign-to-gloss→gloss-to-text is one of the best translation protocols, where instead of training a network for text-to-text translation from scratch, they provide better translation results for gloss-to-text translation. In our approach, we propose a Sign2Gloss translation protocol network using a modified standard transformer.

### 2.3. Video Analysis and Summarization

Sign language translation takes time to process continuous sign sequences. As a result, incorporating video summarization or video processing techniques into SLT may improve gloss recognition accuracy in the Sign2Gloss translation protocol. Video summarization and video processing, on the other hand, are very common in video recognition and action recognition tasks [50]. The primary goal of video processing is to choose a set of frames to facilitate fast computation while processing lengthy videos. Yao et al. [51] proposed a key frame extraction technique based on multifeatured fusion for processing dance videos in order to recognize various dance motions and steps. Furthermore, a smart key-frame extraction technique was proposed by Wang et al. [52] for vehicle target recognition. This model integrates the scale-invariant feature transform (SIFT) and the background difference algorithm, coupled with the concept of criterion factor K, to significantly divide and categorize the frames into non-mutation and mutation frames. The redundant frames are dissimilar frames that are discarded. However, because it skips a greater number of frames compared to SLT, this method is only appropriate for vehicle recognition. To resolve these missing details in frame extraction methods, Li et al. [53] proposed a new concept called sparse coding for key frame extraction with log-regularizer. This method overcomes the challenges of losing pertinent data while discarding redundant frames while performing key frame extraction. However, this method is unsuitable for complex videos because it strips away high-level semantic information from the video.

### 2.4. Pose-Based Methods for SLT

Human pose-based architecture is not only used for action recognition but it has also been applied to perform specific tasks in WSLR and SLT since the advancement of deep learning. Pose estimation is either performed using a probabilistic graphical model or using pictorial structures [54]. So far, human pose estimation has achieved outstanding results for static or isolated images. However, it underperformed for real-time or dynamic images such as video because of issues with tracking occlusions, motion blur during the transition, and its inability to capture the temporal dependency between the extracted video frames. The poses/skeletal holds positional information of a human body pose and can provide important cues [55]. Using the RWTH-Phoenix 2014 T dataset, a skeleton-based graph convolution network was proposed for end-to-end translation. It used only 14 key points, omitting fine-grained key points in fingers and faces, resulting in poor end-to-end translation performance. However, skeletal-based methods have gained attention in modern research methods since they are independent of background variations. Further, in skeleton-based SLR models, RGB-based skeletal methods outperforms well. To overcome this performance degradation stated in the previous work, Songyao et al. [50] proposed a skeleton-aware multi-modal ensemble for RGB frames, which has 33 key points, including key points in the nose, mouth, upper body, and hands. This framework makes use of multi-modal information and utilizes a sign language graph convolution neural network (SL-GCN) to build embedded dynamics. Further, in another work, maxim et al. [56] investigated the enhancement of recognition performance in SLR models by fine-tuning the datasets. Additionally, the author analyzed whether it is possible to use these models in a real-time environment without GPU.

Yang et al. introduced the graph convolution neural network model to deal with the temporal dependency among extracted frames. Followed by him, many others proposed various methods for pose estimation, such as the GCN-BERT method [38], key point extraction methods using open pose [57], action structured graph convolution networks [58], and MS-G3D for spatial–temporal graphical convolution networks.

The pose-based approach proposed by Youngmin et al. [57] introduced video processing and key point extraction techniques. These techniques aided in frame selection and key point extraction for precise body movement and location. Sign-to-text translation protocol was used in this pose-based approach. However, direct translation from sign language video to spoken sentence produced no good results. In addition to these methods, automatic sign language translation is possible by merging the NLP transformers and computer vision. For such tasks, the video-transformer network was proposed by Coster et al. [59]. However, these transformer networks require a huge amount of labelled data corpus to fine-tune and train ASLR models. This method is evaluated using the large-scale annotated Turkish Sign Language data corpus that eliminates the need for a large, annotated data corpus.

## 3. Materials and Methods

In this section, we discuss the baseline methods for our proposed Sign2Pose Gloss prediction transformer architecture that efficiently predicts gloss words from dynamic videos. Identifying the explicit boundaries of sign words from sign videos is a practical difficulty faced by CSLR/SLT systems. Though many techniques have been proposed earlier to solve the end-to-end translation model for efficient mapping of predicted words with the target sentence, the existing systems do have some snags. Intermediate gloss prediction substantially increases the translation outcomes of SLT systems. Therefore, we propose a novel method for efficient gloss prediction using a Sign2Pose Gloss prediction transformer that significantly identifies the intermediate gloss for the given input video sequence. Initially, the system is validated using the WLASL [60] dataset for word-level gloss videos using a sign-to-gloss network translation protocol. As stated in Section 2.1, the proposed model can enhance the efficiency of a two-stage SLT system, reducing the need for a large, annotated vocabulary. Furthermore, it is not required to tune and train a model from scratch when using this proposed word-level gloss prediction transformer; it can be employed as a pre-trained gloss network in two-stage SLT. The methods are subdivided into four phases, namely (i) key-frame extraction, (ii) pose estimation from key-frame, (iii) pre-processing, and (iv) pose normalization. This section briefly elaborates on the key components and steps of the proposed Sign2Pose Gloss prediction transformer.

### 3.1. Dataset Description

We have a wide variety of gestural corpora because sign language is not a universal language. For example, the Chalearn synthetic hand [61] dataset contains realistic 3D human male hand gestures, InterHand 2.6 M [62] is a 3D representation of interacting hands, and the TheRusLan [63] data corpus contains 22,200 audio samples with text annotations. The AUTSL [64] data corpus is a large corpus multi-modal Turkish Sign Language dataset with 226 signers and 38,336 isolated sign video samples. MS-ASL [62] data corpus is a massive corpus with 1000 signs performed by 200 signers in real-world environments. Because we plan to concentrate on the entire body posture to capture the most precise details in the body posture for gloss prediction, we opt for the word-level American Sign Language dataset for our experiments. We train our model using the large-scale signer-dependent word-level American sign language (WLASL) publicly available benchmark dataset [46]. The gloss videos for the above-mentioned dataset are collected from multifarious public websites that hold the gloss annotations for dialects in ASL along with the details of metainformation such as bounding box, temporal boundary, and signer diversity. The average length of videos in the dataset is around 2.41 s. The sign instances are performed by 119 native American signers. Initially, the collected data from multiple public resources planned for tutoring SL led to diversity in signing dialects, styles, and backgrounds suitable for real-time sign language classification. This dataset is categorized into 4 subsets based on different vocabulary sizes such as WLASL 100, WLASL 300, WLASL 1000, and WLASL 2000. These four subsets are grouped by choosing the top K glosses where K = {100, 300, 1000, 2000}. A detailed description of the dataset is briefed in Table 2.

### 3.2. Key Frame Extraction Technique

The dynamic sign word video has multiple video frames with multiple repeated gestures and transition phases between the successive gestures after extraction. This method retains the best representations of shots from extracted frames and discards the redundant frames. Thus, processing all such extracted frames requires a high-power computational system and takes a huge amount of computational time. Therefore, we propose a key frame extraction method using a modified histogram difference algorithm method for discarding unnecessary frames to efficiently boost the performance of the proposed Sign2Pose Gloss prediction transformer for dynamic sign word videos and overcome the timing overhead and computational complexities. The main objective of this method is to decide the specific key frames from actual frames for each signed word that are notable in terms of different gesture positions and thereby disposing of the unwanted poses or gesture positions and transition phases.

We divide this key frame extraction into two phases. In our first phase, we extract the frames from the given input video in a successive manner and then calculate the threshold with mean and standard deviation after applying the modified histogram difference algorithm. The distance between the current and the difference frame is calculated using the Euclidean distance algorithm. After measuring the distance between the frames, the mean and standard deviation is calculated. In our next phase, the threshold value denoted as “*T_h_*” is set, and the measured distances are compared with the threshold.

Let us denote the input video as *“I”,* and the frames are represented as “*f*”. Initially, the frames extracted from the input video are RGB frames. Then, RGB frames are converted to grayscale frames to compute the absolute difference between the frames using the absolute difference algorithm. Therefore, let the RGB frames be denoted as “*f_RGB_*”, grayscale frames are denoted as “*f_GRAY_*”, histogram difference is assumed as “*H_diff_*”, and ‘ℕ’ denotes the number of bins in the histogram.

(1) $$H_{diff}\left(t\right)=\left|\overset{\mathbb{N}}{\underset{j=0}{\sum }}H_{\left(t-1\right)}\left(j\right)-H_{\left(t\right)}\left(j\right)\right|.$$

After computing the difference, we apply mean and standard deviation where “μ” is used as a symbol for mean calculation, and “σ” denotes standard deviation. The distance between the successive frames is calculated using the Euclidean difference algorithm “*E_d_*”.

(2) $$E_{d}\left(p,q\right)=\sqrt{{\left(p_{1}-q_{1}\right)}^{2}+{\left(p_{2}-q_{2}\right)}^{2}}.$$

Let “*p*” and “*q*” be the two points in a frame, and let the coordinates of “*p*” be (*p*_1_, *p*_2_) and “*q*” be (*q*_1_, *q*_2_). For “*n*” dimensions, the formula can be more generalized as follows:

(3) $$E_{d}=\sqrt{\overset{n}{\underset{i=1}{\sum }}{\left(p_{i}-q_{i}\right)}^{2}},$$

where “*n*” denotes the dimensions and *p_i_* and *q_i_* are the data points. After computing the Euclidean distance, the threshold value is set. To set the threshold value, we must perform *H_diff_* and then calculate the mean and standard deviation.

(4) $$T_{h}=\;\mu \;+\;\sigma ,$$

(5) $$T_{h}=\;\varphi ,$$

where “φ” is used to represent the combined value of mean and standard deviation. After setting the *T_h_* value, we compare the measured distance between the consecutive frame and the threshold value, and the choice between the key-frame “*K*” and the redundant frame “*R*” is conducted. The elements in key-frames are denoted as “k_N_”, and the elements of redundant frames are represented as “r_M_”. Then, the extracted key frames are provided as inputs for pose extraction for gloss prediction. The detailed steps for key frame extraction are provided in Algorithm 1.
**Algorithm 1.** Key-frame extractionInput:Let *I* be the input sign video *I*$\in \left(1\;to\;N\right)$
*I_i_….I_N_* Let *n* be the number of frames in *I_i_*Output:Set of key-frames *f_key_*: *f*_key_ {1 to m} where m < n 1for *f_RGB_* in n (frames): 2Convert RGB frames into grayscale frames *f_RGB_ → f_GRAY_*3 Compute histogram difference *H_diff_* between successive frames using Equation (1) 4Calculate mean μ and standard deviation σ of the *H_diff_*5Compute threshold value “*T_h_*”:6 Calculate the Euclidean distance *E_d_* using Equation (2)7 *f_GRAY_* ={elements of *K* and elements of *R*} “*R*” denotes the set of redundant frames Such that, *K* = {k_1_, k_2_, k_3_,…k_N_} *R* = {r_1_, r_2_, r_4_,...r_M_}8for I in n:9if *E_d_ > T_h_*:10*R\K* = {r_M−1_} Element obtained belongs to set of redundant frames but not to set of key-frames Add the frames to the set *f_key_*
11else12Discard the frame13Repeat steps 1 to 12 for the entire dataset, and once completed, discarding redundant frames stops the process.

### 3.3. Pose Estimation from Key-Frame

Human pose estimation (HPE) refers to representing the orientation of the human body in a graphical format. In other words, locating human body parts and joints using computer vision in images or video. Initially, before the deep learning era, human pose estimation was performed by recording an RGB image using optical sensors and kinect sensors to detect the human pose or an object. The three most common types of human models are skeleton-based, volume-based, and contour-based. Skeleton-based HPE is the most preferred and frequently followed method since it is flexible with stability in the joint locations and orientations. For instance, ankles, wrists, knees, elbows, shoulders, fingertips, etc. There are various standard frameworks for pose estimation. Pischulin et al. proposed a deep-cut algorithm [65] for multi-person pose estimation with joint objectives. This method first locates the person’s joints using integer linear programming. The method identifies the joints much more precisely though occlusions appear from person to person, but the process is extremely complex and time-consuming. Further, various other frameworks using deeper cut algorithms [66], PoseNet [67], and OpenPose [68] are used for HPE. In our proposed framework for pose estimation, we use standard pose estimation of vision API for locating the head, body, and hand landmarks from each set of “K” video frames. The landmarks obtained are all 2D and relative to the frames. Its coordinate values for the top right side to the frame are [1, 1], whereas the bottom left corner is denoted as [0, 0]. We use a vision image classifier to spot the presence and absence of individual landmarks or objects. If an object is absent in the relative frame, then the coordinates are represented by 0 and vice versa.

### 3.4. Pre-Processing

**Figure 2:**
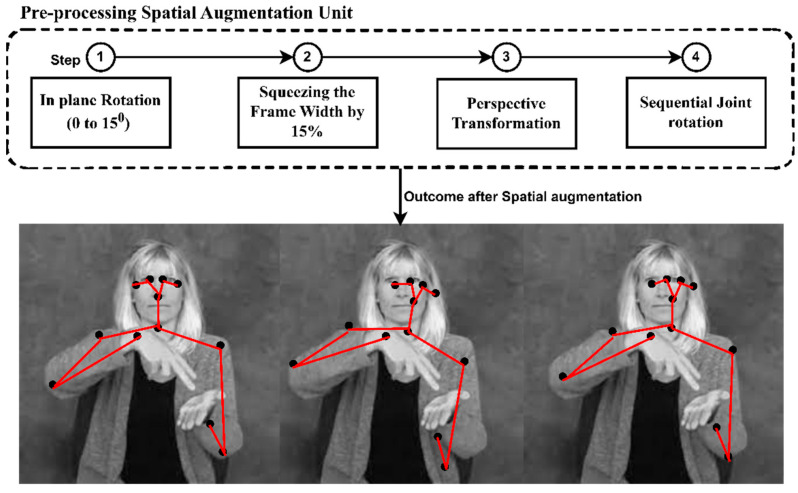
Illustrating the augmentation techniques applied to a single frame while pre-processing.

After acquiring landmark coordinates in pose estimation, we opt for a pre-processing technique to efficiently enhance the system’s generalizing ability. For the system to adapt to different datasets and develop a versatile response, we employ spatial augmentation while training the skeleton data points. Further, the choice of the parameters is random, and they have maintained rationale throughout the signing space for every frame. Additionally, this spatial augmentation technique overrides the overfitting issue. Figure 2 depicts the steps involved in pre-processing and the outcome of augmentation applied on single frames.

The initial step in spatial augmentation is applying in-plane rotation to each frame whose angle of rotation, denoted by “*θ*”, lies between 0° to 15°. Therefore, by applying in-plane rotation, the plane is mapped to itself for a particular rotation and does not remain fixed. Perhaps all the vectors in the planes are mapped to other vectors in the same plane by rotation. During rotation, the center of rotation confides on the center of the plane (frames) coordinates (0.5, 0.5). For instance, in a 2D image, the position of point P is represented by the coordinate (P_x,_ P_y_), and the numerical representation of point P in a plane is anonymous until we define a reference coordinate. Once the origin is fixed, the extents of point P from its x and y axis from the origin are its coordinates (P_x,_ P_y_).

Followed by in-plane rotation, the next step we carry out is squeezing the frames on their horizontal sides by setting random proportions ranging up to 15% of the original frame width w_1_(right side) and w_2_ (left side). Once the squeezing is set, the joint coordinates are recalculated concerning the newly set plane. The third step is perspective augmentation, where the sign video is recorded with a minor shift in angles of inclination applied to the signing video. This method helps the system become accustomed to images with different angles and builds the system’s robustness. Like human vision, which can locate and identify an object at any distance and any angle of inclination or distance, perspective augmentation helps the model recognize the same 2D or 3D object on two different projective planes.

By applying perspective transformation, the joint coordinates are made to project on a new plane with spatially defined projection with a slightly inclined angle. The proportion and adjustment to the right and left sides of the single frame are picked randomly by uniform distribution with an interval of [0, 1]. The detailed steps of sequential joint rotation are explained in Algorithm 2.
**Algorithm 2.** Sequential Joint Rotation1Input image *I_in_, x,* and *y* standard coordinates2Initialize center point of the frame as C*_mid_*3Fix C*_mid_* = 0.54Rotate frame *f_rot_* according to C*_mid_, and [x,y]* Standard Rotation Matrix is given as R $R=\left(\begin{array}{cc} \cos\theta & -\sin\theta \\ \sin\theta & \cos\theta \end{array}\right)$
5*f_rot_ with*$\;\mathrm{respect}\;\mathrm{to}\;\mathrm{standard}\;\mathrm{coordinates}\;[xy]\;\left(\begin{array}{cc} \cos\theta & -\sin\theta \\ \sin\theta & \cos\theta \end{array}\right)\left(\begin{array}{c} \left(x-0.5\right)\\ \left(y-0.5\right) \end{array}\right)$ then the moved state is denoted by *x’ and y’* *x’ = (x −*0.5) cosθ – (*y* − 0.5) sinθ + 0.5 *y’*
=(y− 0.5) cosθ + (*x* − 0.5) sinθ + *0.5* *f_rot_(x’y’) = (x − *0.5*)* cosθ – (*y* − 0.5) sinθ + 0.5, (*y* − 0.5) cosθ + (*x* − 0.5) sinθ + 0.56Angle of rotation *θ ≤* 15°7Generate random moving state *S_m_* based on *θ* and uniform distribution8Within the range of C_mid_, move *x* based on *S_m_,* then *y* based on *S_m_* to calculate *S_m_^’^* to obtain a new range of *x* and *x’*, *y* and *y’* I_Augmentation_ = Augment (I_in_, *x*, *y*) I_Augmentation_’ = Augment (I_in_, *x’*, *y’*)9Calculate recognized image *I_obs_* and measure the Euclidean distance *E_d_*10**if** *E_d_(I_obs_, C_mid_) ≤ E_d_(I_obs_’, C_mid_)***then** Improve the recognition accuracy **else** stop

### 3.5. Pose Normalization

Body proportion differs from person to person. Not only this, but positional properties, such as camera distance, capturing angle, angle of rotation, motion transfer, head, face, hand, and palm orientation, etc., vary from signer to signer. Further, input landmark coordinates are associated with values relative to the frame. This leads the model to learn more irrelevant spatial features than the performed sign. In such cases, training and fine-tuning the model will be time-consuming. To overcome this issue, we use the normalization technique, where all such body proportions, distance from the camera, positional properties, motion transfer overheads, and orientation are precluded. Inspired by SL linguistics [69] regarding the use of signing space with body landmarks, we use a 3D space in the signing space in front of the signer and their immediate surroundings. We take the area slightly above the signer’s waist, reaching slightly above the signer’s head, covering the two loosely bent elbows with projected body landmarks to identify the sign.

**Figure 3:**
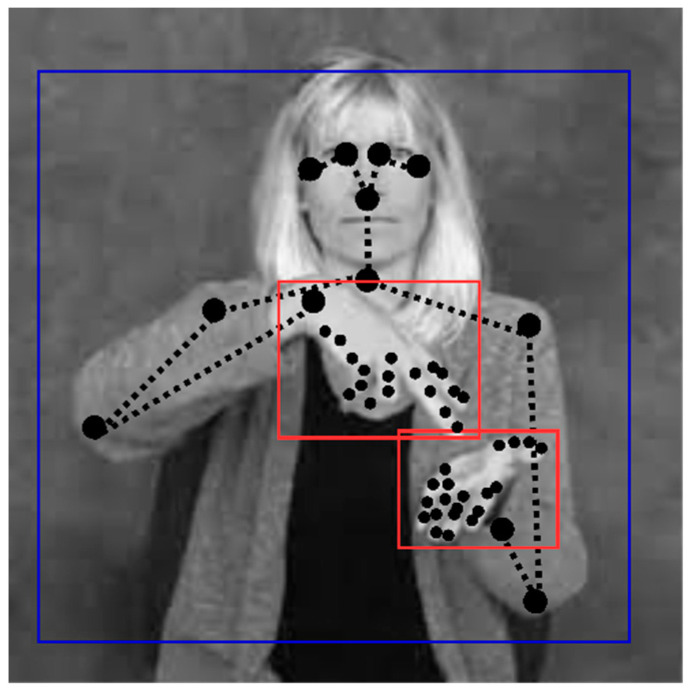
Sample visualization of normalized pose using YOLOv3.

In our previous pre-processing step, we applied augmentation techniques to efficiently enhance and bring versatile recognition for different body proportions, orientations, and tilting angles. Though our model is efficient towards generalized input, without normalization, the system picks inappropriate spatial features from the signs performed. So, we use normalization using YOLO version 3 for object detection and pose normalization using anchor boxes. We define a signing space based on a head portion with 7 head units wide and 8 head units high where its horizontal center lies with a nose. Additionally, the vertical side of the anchoring box is fixed, considering the left eye with 0.5 head units upright and 6 units below for the bottom edge. We have two other anchoring boxes for tracking the hand orientations and their shape, which enables the model to target the hand orientations and their corresponding signs, eliminating all other insignificant spatial features relative to the frame. Figure 3 shows the visualization of the normalized pose using YOLO v3 for an independent frame.

To calculate the anchor box and its normalization, we need to rescale them between 0 and 1 by dividing the image width by its height. The bounding box network format is (x, y, width(w) and height(h), confide). In YOLO v3, the predicted output coordinate of the anchor box is normalized relative to the grid and input image. We do this because we have diverse signers, and among such a diverse dataset, the model should detect the sign performed. The confide value is set to 0.5, and depending on the confide value, the object is detected. As we know, the annotation coordinates are (*X_max_, Y_max_, X_min_, Y_min_*), considering (*X*_1_, *Y*_1_) as *X* and *Y* coordinates of the top left corner of the bounding box. (*X*_2_, *Y*_2_) are the X and Y coordinates of the bottom right corner of the bounding box and (*X*_c_, *Y*_c_) are the center *x* and *y* coordinates of the bounding box.

Where

(6) $$\left(X_{max},Y_{max}\right)=(X_{1,}Y_{1}),$$

(7) $$\left(X_{min},Y_{min}\right)=(X_{2,}Y_{2}),$$

(8) $$Normalized\;X_{min}=\left(X_{min}+w/2\right)/W_{img}),$$

(9) $$Normalized\;Y_{min}=\left(Y_{min}+h/2\right)/H_{img}),$$

(10) $$Normalized\;width\;\left(w\right)=w/W_{img},$$

(11) $$Normalized\;height\;\left(h\right)=h/H_{img}.$$

The bounding box coordinates, width, and height lie between a particular location of the grid cell, so they balance between 0 and 1. Furthermore, the sum of the square error is calculated only when the object is present.

## 4. Proposed Architecture

**Figure 4:**
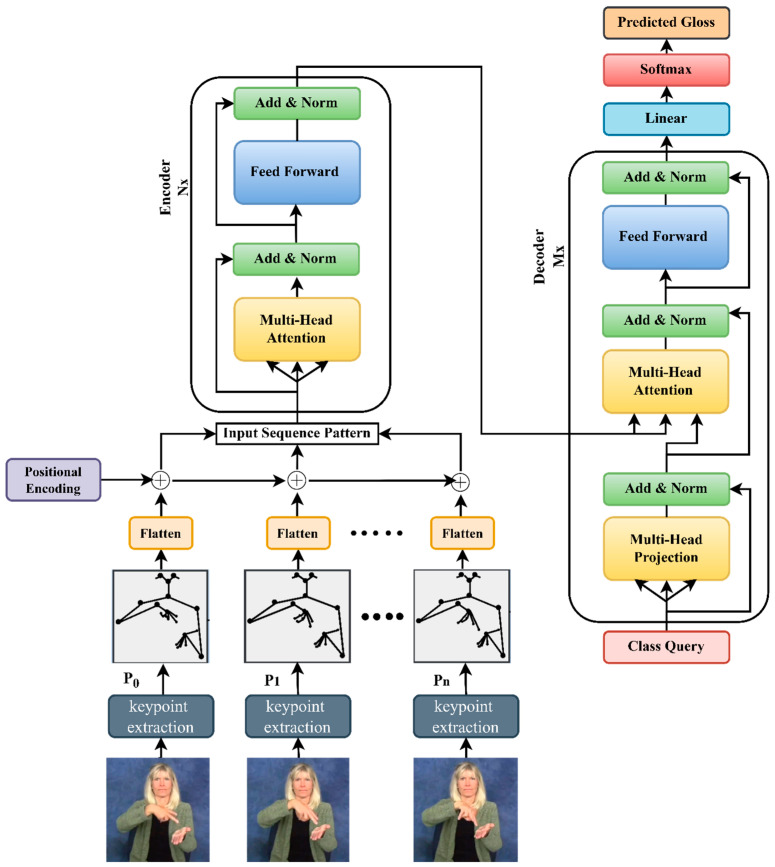
Proposed architecture of the Sign2Pose Gloss prediction transformer.

The sequence of movements in body parts provides a lot of information in sign language. Moreover, in our literature study, we analyzed that pose sequences are outstanding records in recognition and detection since the model stays focused on features in the pose images rather than looking into inappropriate components such as background, lighting, and so on. In our proposed architecture, we used the Sign2Pose Gloss prediction transformer, which is a slightly modified version of the transformer with attention [31]. The input to our proposed transformer model is a normalized pose sequence with a 108-dimensional pose vector and 54 joint locations. The Sign2Pose Gloss prediction transformer uses attention skillfully. Figure 4 depicts the entire architecture of our Sign2Pose Gloss prediction transformer. We have an encoder and a decoder layer where the model first translates the sequence of sentences and then applies vectorization, and finally, with the attention layers, transforms them. In our model, we use learned positional encoding rather than spatial positional encoding to define the actual semantics of the sentences and words. Furthermore, we add the positional encodings with 108 dimensions to the individual pose vectors. By adding the learned encodings to the individual pose vectors, we obtain a sequence of input vectors to fetch as input to the encoder layer. There are six layers in both the encoder and decoder, with nine head units in the self-attention module and an input dimension of 108, followed by 108 hidden dimensions and a feed-forward dimension of 2048. As per the standard transformer model, there are two self-attention and feed-forward networks.

The standard transformer decoder architecture has query, key, and value vectors as output vectors for each word. For instance, if the input sequence vector to the encoder is “*It’s too sunny today*”, then the input embeddings present in the sentence are four, and for each input vector/input word, we calculate three output vectors such as query, key, and value. In the above-stated example, we have “*n* = 4” words. Thus, for “*n*” words in a sentence, there are nQueries, keys, and vectors to be calculated. In our case, we are proposing a Sign2Pose Gloss prediction transformer for gloss prediction. We use the word-level American sign language dataset, and the sequence vector processed through the entire encoder and decoder process will be a single element. For this purpose, we have one query at the input of the decoder, and that query is called class query since it decodes the class of sign. Since there is only one element to be processed through the entire multi-head projection module present in the decoder, the attention has no influence on key and value vectors, and the SoftMax present in the attention model is always “1”. Hence, we calculate the input vector in the value space and there is no requirement for key and query calculation.

After the processing elements pass through the multi-head attention module in the decoder layer, the vectors are concatenated and processed by the linear layer using logit vectors. In this linear layer, we provide the class query input where the confidence of each class is calculated using the SoftMax activation.

## 5. Experiments

For our experiments, we used a dataset of American sign language at the word level. There are four subsets of datasets, as described in Section 3.1, and we utilized every subset separately for our experimental evaluation. WLASL 100, WLASL 300, WLASL 1000, and WLASL 2000 comprised the evaluation dataset. The suggested transformer model for gloss prediction was trained using the aforementioned datasets. The datasets were split in the ratio of 85:15, out of which 15% of the dataset was used for testing and from 85%, 70% was used for training, 15% for validating, and the remaining 15% for testing. We proposed a novel method to build a robust model which is more flexible in recognizing similar signs, learning different dialects, and coping with different environments with different signers. We have applied a key-frame extraction module to discard the redundant frames and implied augmentation technique. After augmentation, we used YOLO version 3 to normalize the pose vectors in such a way that our system is free from overfitting. With all these pre-processing steps, we input a normalized pose image with all landmarks to the proposed transformer architecture. In addition to the original context of the sign, horizontal flipping was set to 0.5 randomly for all the normalized frames. The details of the parameter tuning of our model are stated in Table 3.

The proposed Sign2Pose Gloss prediction transformer was implemented using TensorFlow in the Anaconda Software tool. The query, key, and value vectors in the standard transformer models were slightly modified to discard the unnecessary computations inside multi-head attention in the decoder module that may occur due to the flow of class query through the module. We run our experiment for 300 epochs, and the learning rate was set to 0.001 with the weighted decay 10^−4^, and momentum was set to 0. We used a stochastic gradient descent optimizer, and the weights were initialized using uniform distribution ranging (0, 1). This range was randomly fixed, and we used the cross-entropy loss function to score the models’ performance in terms of correct gloss prediction.

## 6. Results and Discussions

We evaluated our pose-based proposed model on all the subdivisions of the publicly available word-level American sign language datasets. As mentioned in Section 3.1, Table 2, WLASL datasets have Top *K* classes, where subsets/classes WLASL 100, 300, 1000, and 2000 are based on the number of videos. For instance, the first subset of WLASL has 100 classes, and each class represents a particular gloss video with different instances performing the same gloss under the same class category. We compared our results with previous pose-based and appearance-based models to evaluate the models’ performance and state-of-the-art outcome achieved by our model in the same dataset. For ease and a prospective comparison of the advancement of the primary data representation streams for SLR, the findings of appearance-based techniques were also considered. Table 4 summarizes the previous pose-based and appearance-based models experimented on subsets of WLASL and other datasets.

**Figure 5:**
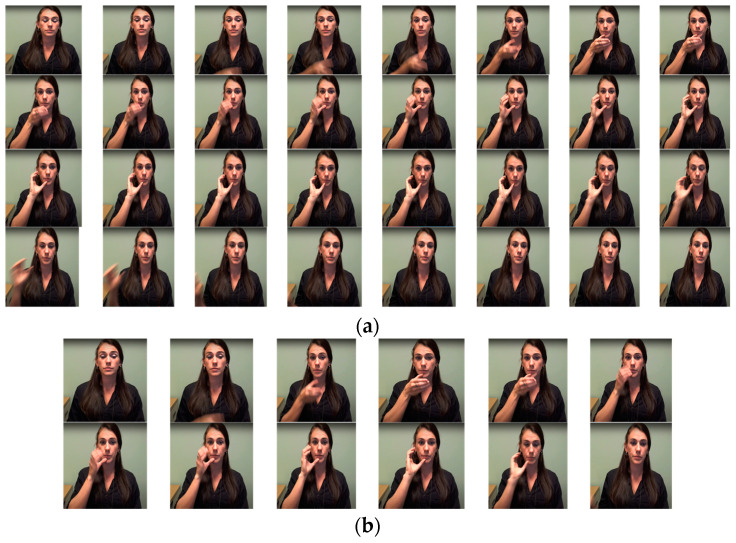

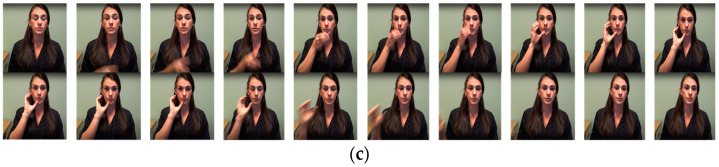
Sample images of key-frame extraction for the Gloss “Drink” from the WLASL 100 dataset (**a**) sample of extracted frames for the mentioned gloss. (**b**) Discarded redundant frames. (**c**) Preserved key-frame sample from extracted frames.

As discussed in Section 3.2, sign word videos have multiple frames. In our baseline model, after the extraction of frames from the sign video, we used the key-frame extraction technique to preserve key-frames and discard irrelevant frames. This method reduces processing time complexity and improves the clarity of critical frame predictions for gloss. Further, to make the system more reliable for generalization, we used special augmentation techniques, as mentioned in Section 3.4, and we used YOLOv3 to normalize the pose vectors to fetch as input to the slightly modified standard transformer model proposed by Camgoz et al. [31]. The use of YOLOv3 not only boosts the systems gloss prediction, but our method also overrides overfitting issues. Figure 5a shows an example of the key-frame extraction for the word “Drink”. Frames that were pulled out for the gloss “Drink” had transitional frames between repeated and idle frames. We applied a modified histogram difference algorithm and Euclidean distance algorithm to extract the key-frames and discard the redundant frames, as discussed earlier in Section 3.2. Figure 5b shows the sample of the discarded frames eliminating the blurred, idle, and transitional frames and Figure 5c shows the extracted key-frames using Algorithm 1 in Section 3.2.

Through this technique, we were able to achieve top 1% accuracy of 80.9 in WLASL 100, 64.21% in WLASL 300, 49.46% in WLASL 1000, and 38.65% in WLASL 2000. However, we contrasted our model with models that are based on both poses and appearances. Our suggested method outperforms the prior state-of-the-art pose-based approach on the WLASL100 by 17 percentage points, attaining 80.9% in top 1% accuracy. On the WLASL300 subset, we also created a state-of-the-art result of 64.21% accuracy, outperforming the prior one by 20 percentage points.

**Figure 6:**
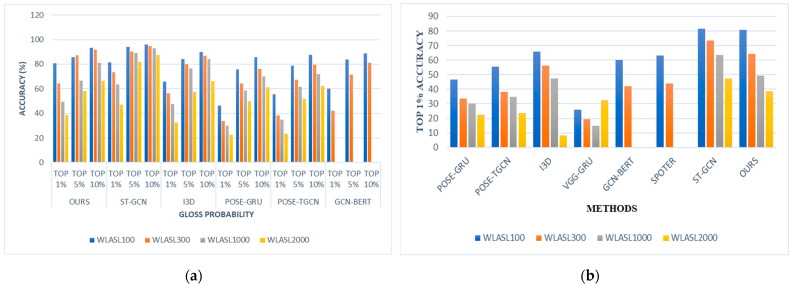

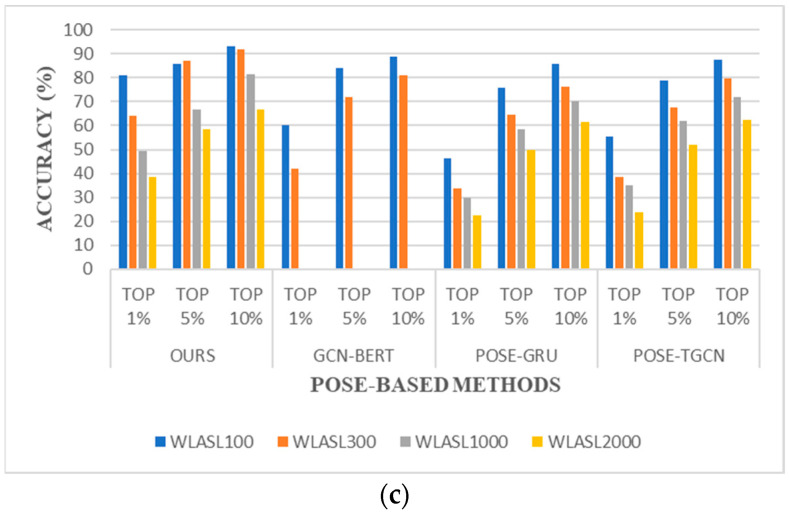
Performance analysis of proposed work with existing appearance and pose-based models. (**a**) Graphical representation comparing our approach with the pose-based as well as appearance-based model. (**b**) Comparing top 1% recognition accuracy on both pose-based and appearance-based models; (**c**) comparing top K macro recognition accuracy on pose-based models.

**Figure 7:**
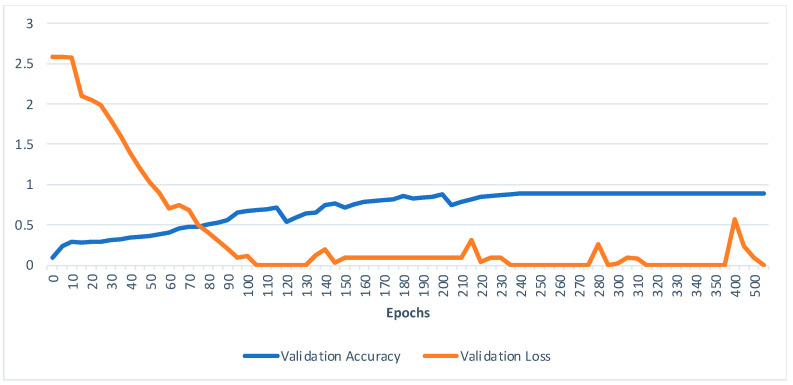
Validation accuracy and validation loss of our model.

From Figure 6a,b, it is observed that the appearance-based models surpassed pose-based models. Though these appearance-based models (ST-GCN and I3D) outrun our model, we contend that these results come at a substantially higher computational cost owing to the dimensions, which are limited in our system even when coupled with the pose estimation framework. In Figure 7, we observe the model’s ability to predict top 1% gloss prediction accuracy during validation with the test samples, and the loss accuracy determines the predicted number of incorrect glosses by our models. The previous pose-based model underperforms in recognizing different words with similar signs, such as “man”, “woman” “read”, “dance”, “wish”, “hungry”, “cold”, “hug”, “circle”, “turn-around”, “runny nose”, “head-cold”, which slightly vary in their hand orientation. From observing Figure 6c, it is analyzed that our model has seamless improvement top 1% validation accuracy of 80.9% when compared with other pose-based models since the proposed Sign2Pose Gloss prediction transformer uses the hand-crafted input feature representation of body and hand stance that already has sufficient information to decode the notions needed for sign language compared to other appearance-based models. As a result, it needs a much smaller training set to obtain adequate results.

From Figure 7, it is observed that the model starts to converge from 150 epochs and attains its maximum top 1% macro recognition accuracy by 220 epochs, and the model performs consistently after 240 epochs attaining 80.9% accuracy as top 1 class accuracy for WLASL 100. We tested the model after training it using a fixed dataset split of 15%. Table 5 shows the compositions of techniques and their top 1% recognition accuracies (%) on four subsets of the WLASL dataset. Our model uses a standard pose estimation algorithm from apple vision API and YOLO V3 for extracting the bounding and anchoring box for the hand. Using this technique, our extraction method is strong and effective, especially in near and different sign viewpoints.

**Figure 8:**
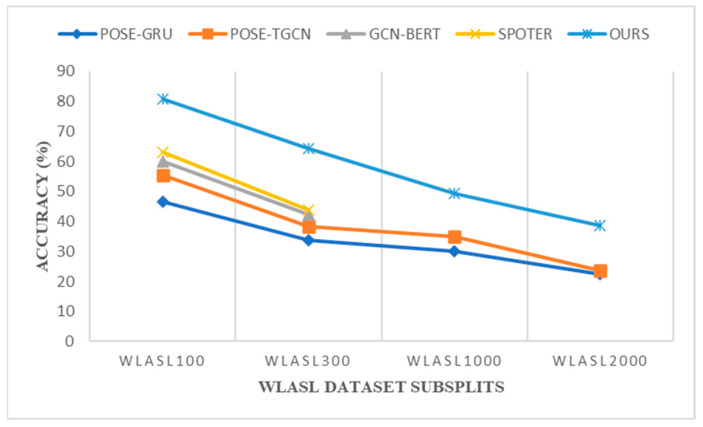
Comparison of the pose-based approaches’ top 1 accuracies (%) and scalability on four subsets of the WLASL dataset.

Figure 8 illustrates how our proposed initiative, which employs hand-crafted feature engineering techniques before the gloss prediction transformer, consistently increased its recognition accuracy in the top k classes WLASL datasets by about 17% compared to prior state-of-the-art models. We have provided a gloss prediction example from our model for ease of comprehension. Although our model is pose-based, we have taken into account key retrieved RGB color mode frames. Table 6 shows that 84.8% of all occurrences presented under this gloss class group were properly predicted, including the tiniest variation, “Baby”. Additionally, in contrast to previous pose-based models, the average inference time during validation was 0.03 s. Our approach also performed well on datasets with few instances. In comparison to the previous pose-based architecture, the top 5% and top 10% recognition accuracy for all the WLASL model subsets exhibited a consistent growth of 4 to 10%. In comparison to appearance-based systems such as I3D and ST-GCN, our Sign2Pose Gloss prediction transformer proved to be significantly more suitable for applications in the real world in terms of model size and speed.

Figure 8 shows that our suggested strategy consistently beat all other existing models, regardless of the size of the datasets, with an improvement of 17 to 20% over the methods now in use. In the appearance-based model, they face difficulty in predicting gloss words with slight variations in their hand orientation. Additionally, difficulty arises in detecting the bounding box when the sign is performed by the signer in the side view angle. When considering pose-based models, prior approaches could not fully benefit either from normalization or optimizations other than the regular ones carried out to visual data. As our model relies on body pose sequence representations as its foundation, we used insights from sign linguistics to develop a solid normalization methodology using YOLOv3 as well as fresh data augmentation methods tailored to sign language.

## 7. Conclusions

A real-world challenge for CSLR/SLT systems is determining the clear boundaries of sign words from sign videos. Although alternative techniques have been put out in the past to resolve the end-to-end translation model to ensure efficient mapping of anticipated words with the target sentence, there remain some limitations with the current systems. The performance of the SLT systems’ translation is significantly improved by intermediate gloss prediction. In this paper, we proposed a novel approach for gloss prediction using the Sign2Pose Gloss prediction transformer. Instead of relying on pre-trained models to tackle gloss prediction, we used hand-crafted techniques for pose feature extraction that not only predicted gloss more precisely but also decrease processing overheads. With the help of a SignPose2 Gloss prediction transformer, we provided a novel approach for effective gloss prediction that considerably identifies the intermediate gloss for the provided input video sequence. For efficient gloss prediction by our proposed architecture, we used a modified HD algorithm for key-frame extraction to differentiate key frames from redundant frames. We also employed the Euclidean distance algorithm to sort the key-frames and redundant frames based on the threshold value. Further, we equipped our model with augmentation steps, making it more adaptable to any real-time dataset. YOLO v3 was then applied to the pose vectors to detect the precise movements of the hand. The use of YOLO v3 brought a drastic improvement of about 15–20% in our model accuracy which surpassed all the current pose-based methods. In all subsets of the word-level ASL data corpus, our model produced more state-of-the-art results than other pose-based approaches. In the future, we plan to amplify our model with modern skeleton frameworks that allow for further efficient continuous sign translation from intermediate gloss representations. We will also evaluate the proposed work and future frameworks using large-scale annotated data corpora such as AUTSL, MS-ASL, and others.

## Figures and Tables

**Table 1:** Summary of existing methods for gloss prediction using two-stage SLT.

Ref.	Translation Type	Technique for Gloss Prediction	Dataset	Performance Metric	Remarks
[38]	Sign2Gloss2Text	Graph convolution network (GCN) and bi-directional encoder representations from transformer (BERT)	WLASL	88.67 at top 10% accuracy on 100 gloss recognition	Image-based feature extraction enhances the performance of the model.
[44]	Sign2Gloss2Text	Human key-point estimation	KETI sign language	BLEU4—65.83 (Key points: Hand, body)	Performance would improve on improving key-point detection
[45]	Sign2Gloss2Text Gloss2Text	Spatial-temporal transformer and spatial-temporal RNN	Phoenix 2014T	BLEU4-24.00	Dataset is restricted to the weather forecast
[46]	Sign2Gloss2Text	Temporal graph convolution network (TGCN)	WLASL	62.63% at top 10 accuracy on 2000 gloss recognition	Labelling a large number of samples requires advanced deep algorithms to pave the way from word-level to sentence-level annotations
[47]	Sign2Gloss2Text	Context-aware GAN, temporal convolution layers (TCL), and BLSTM	Phoenix 2014T, CSL, and GSL signer independent	23.4%, 2.1%, and 2.26% WER, respectively	Complexity and data imbalance in GAN network
[48]	Sign2Gloss2Text	Transformer	WLASL100, WLASL300, and LSA 64	63.18%, 43.78%, and 100% recognition accuracy	Shows better outcomes on even smaller datasets
[49]	Sign2Gloss2Text	Intensity modifier	Phoenix 2014T	BLEU1-26.51	Lacks spatial and temporal information for black translation and lack of proper evaluation metrics.

**Table 2:** WLASL dataset description.

Categories	Content	Type	Glosses	Samples	Mean (Avg. Instances/Class)	Signers
WLASL 100	Video with Aligned Sign/Sentence with text and Gloss	RGB	100	2038	20.38	97
WLASL 300			300	5117	17.1	109
WLASL 1000			1000	13,168	13.16	116
WLASL 2000			2000	21,083	10.54	119

**Table 3:** Hyperparameter specifications.

Hyperparameter	Tuning Details
Pose vectors	108
Encoder layers	6
Decoder layers	6
Input and hidden dimension	108
Feed Forward dimension	2048
Learning rate	0.001
Weighted decay	0.0001
Optimizer	Stochastic Gradient Descent
Epochs	300

**Table 4:** Summary of different models experimented using WLASL datasets with and without augmentation techniques for prospective comparison of the proposed model.

Model and Dataset	I3D [70]	Pose-GRU [70]	Pose-TGCN [70]	GCN-BERT [38]	ST-GCN [71]	SPOTTER [48]	OURS
Appearance-based	✓	✕	✕	✕	✓	✕	✕
Pose-based	✕	✓	✓	✓	✕	✓	✓
Augmentation	✓	✓	✓	✕	✓	✓	✓
WLASL 100	✓	✓	✓	✓	✓	✓	✓
WLASL300	✓	✓	✓	✓	✓	✓	✓
WLASL1000	✓	✓	✓	✕	✓	✕	✓
WLASL 2000	✓	✓	✓	✕	✓	✕	✓
Other datasets	✕	✕	✕	✕	✓	✓	✕

**Table 5:** Performance analysis on top 1% macro recognition accuracy of proposed Sign2pose Gloss prediction transformer with other pose-based state-of-the-art models.

Pose-Based Models	WLASL100 Top-1% Accuracy	WLASL300 Top-1% Accuracy	WLASL1000 Top-1% Accuracy	WLASL2000 Top-1% Accuracy
POSE-GRU [46]	46.51	33.68	30.1	22.54
POSE-TGCN [46]	55.43	38.32	34.86	23.65
GCN-BERT [38]	60.15	42.18	-	-
SPOTER [48]	63.18	43.78	-	-
Our’s	80.9	64.21	49.46	38.65

**Table 6:** Top 1% accuracy of the predicted gloss matching ground truth label.

Extracted Key-Frames						Top 5 Predicted Gloss	Top 1% Accuracy	Ground Truth
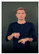	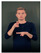	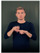	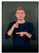	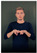	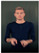	Connect Cut **Chair** Seat Sit	93.6%	Chair
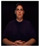	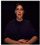	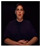	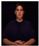	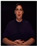	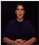	Swing **Baby** Tummy Swaddle Platter	84.8%	Baby
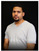	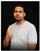	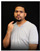	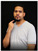	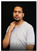	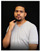	**Neck** Collar Necklace Lip Smash	88.5%	Neck
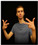	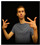	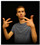	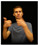	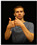	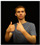	Collide Hit **Match** Unite Relate	90.35%	Match

## Data Availability

The dataset used in this research is publicly available at https://dxli94.github.io/WLASL/ accessed on 1 March 2023.
